# Genome-wide identification of the UGT gene family in foxtail millet and preliminary functional analysis of *SiUGT26* during leaf senescence

**DOI:** 10.3389/fpls.2026.1914896

**Published:** 2026-10-07

**Authors:** Feng Liu, Chao Liu, Xiaoxi Zhen

**Affiliations:** 1 Department of Social Services, Shanxi Agricultural University, Jinzhong, China; 2 College of Agronomy, Shanxi Agricultural University, Jinzhong, China

**Keywords:** foxtail millet, leaf senescence, SiUGT26, SiWRKY71, UGT gene family

## Abstract

Plant uridine diphosphate glycosyltransferases (UGTs) catalyze glycosylation, a pivotal biochemical modification essential for diverse metabolic and physiological processes. Foxtail millet (*Setaria italica*), a drought-tolerant C4 cereal crop, serves as an emerging model for studying abiotic stress adaptation. Here, we identified 173 *UGT* genes (*SiUGT*s) in the foxtail millet genome, unevenly distributed across nine chromosomes and classified into 17 phylogenetically distinct subfamilies. Collinearity analysis revealed nine segmental duplicated duplication pairs under purifying selection, highlighting evolutionary conservation. Promoter analysis identified cis-acting elements associated with growth and development, biotic/abiotic stress responses, and hormonal regulation, suggesting functional versatility of *SiUGT*s. Transcriptome profiling under dark-induced leaf senescence (DIS) revealed 5 up-regulated and 27 down-regulated *SiUGT*s, implicating their roles in senescence regulation. Subsequently, RT-qPCR validated *SiUGT26* as a senescence-responsive gene. Heterologous overexpression of *SiUGT26* in Arabidopsis was associated with delayed leaf senescence and enlarged rosettes compared with wild-type plants. Co-expression analysis suggested that SiWRKY71 may be associated with the regulation of *SiUGT26*. Collectively, this study provides a genomic resource for the UGT gene family in foxtail millet and identifies SiUGT26 as a senescence-responsive candidate gene. These findings provide a basis for further investigation of UGT-mediated regulation of leaf senescence in foxtail millet.

## Introduction

Plants contain a wide variety of low molecular weight compounds, collectively known as plant secondary metabolites (Rai et al., 2016). The synthesis of these metabolites is significantly influenced by key biochemical modifications, such as glycosylation, methylation, hydroxylation, and acylation, all of which are essential for the production of various plant metabolites (Saito et al., 2013). Glycosylation, in particular, is a process that is widespread across numerous organisms, and its mechanisms are highly conserved (Saito et al., 2013; Nagashima et al., 2018; Altanzul, 2023). Glycosylation reactions are catalyzed by glycosyltransferases, which play a critical role in modulating plant hormone activity, endogenous compound metabolism, self-defense responses, and overall plant growth and development (Behr et al., 2020; Tiwari et al., 2016; Gharabli et al., 2023; Song et al., 2018). The UDP-Glycosyltransferase (UGT) family includes many genes that transfer active glycosides from nucleoside sugars to specific sites on proteins, lipid hormones, and other receptors, forming glucoside bonds (Lairson et al., 2008; Speeckaert et al., 2020; Zhang et al., 2020). The most recent CAZY database indicates that the glycosyltransferase superfamily consists of 111 families (GT1-GT114), excluding GT36, GT46, and GT86 from this classification. The GT1 family, known for its catalytic activity in guanosine diphosphate glucose-UDP or uracil nucleoside diphosphate glycosylation reactions, is the largest within this superfamily and has been extensively studied due to its critical biological functions (Vogt and Jones, 2000). Members of the GT1 family feature a conserved 44-amino acid sequence at their C-terminus, termed the plant secondary product glycosyltransferase box (PSPG box) (Li et al., 2001, 2014), which is identified in various species. Arabidopsis thaliana contains 122 UGT genes, while Zea mays and Glycine max possess 148 and 128 UGT genes, respectively (Mamoon Rehman et al., 2016). The UGT gene family has been reported in various plants, including Oryza sativa (Shen et al., 2025), Triticum aestivum (He et al., 2018), Solanum lycopersicum (Qiu et al., 2023), Solanum tuberosum (Ma et al., 2024), and Fagopyrum tataricum (Yang et al., 2024).

Numerous UGTs play a key role in secondary metabolism and morphogenesis throughout plant growth and development. In Arabidopsis, two long non-coding RNAs (NATsUGT73C6) levels affect cell proliferation, thereby influencing the size of individual leaves (Meena et al., 2023). The expression of AtUGT87A2 is developmentally regulated, and its protein products are localized in both the cytoplasm and nucleus. Research has established that AtUGT87A2 influences the flowering time of Arabidopsis through its interaction with the FLC gene (Wang et al., 2012; Wu et al., 2012). A mutation in OsPLS3 causes a deletion in the GT14 glycosyltransferase gene, resulting in the loss of an unidentified functional domain (DUF266). The expression level of OsPLS3 progressively declines in senescent plants, suggesting that OsPLS3 negatively regulates the senescence process (Li et al., 2018). Comprehensive whole-genome analyses in cotton have identified multiple UGTs within its genome. Functional studies have shown that silencing GHUGT116 delays dark-induced leaf senescence (Chen H. et al., 2022). AtUGT76B1 plays a crucial role in systemic acquired resistance (SAR) by modifying N-hydroxy-pipecolic acid (NHP), a key signaling molecule, thus negatively regulating SAR pathways (Mohnike et al., 2021). OsUGT706D2 regulates variations in flavonoid metabolite content, such as alfalfa -4′-o-eugenol ether -7-O-glucoside, and enhances cold tolerance in rice by modulating abscisic acid pathways (Liu et al., 2024). GSA1 encodes UGT83A1 in rice, a UDP-glucosyltransferase that regulates grain size by modulating cell proliferation and expansion through its glucosyltransferase activity toward flavonoids and monolignols (Dong et al., 2020). Recent studies have highlighted the role of UDP-glucosyltransferase71C4 (OsUGT71C4) in cotton seed dimensions, which influence yield. Overexpression of UGT71C4 enhances flavonoid transfer activities, redirecting metabolic fluxes from lignin biosynthesis to increased flavonoid production, resulting in larger seed size (Cao et al., 2024).

Foxtail millet (*Setaria italica*) is recognized for its strong stress tolerance and broad adaptability and has been used as a model crop for studying C4 photosynthesis, abiotic stress adaptation, and cereal improvement (Yang et al., 2020; 2022). Leaf senescence is a complex developmental process involving the decline of photosynthetic capacity and nutrient remobilization (Guo et al., 2021). Although UGT gene families have been studied in several plant species, little is known about the UGT family in foxtail millet, especially their expression changes during dark-induced leaf senescence.

In this study, we identified 173 UGT genes in the foxtail millet genome and analyzed their chromosomal distribution, phylogenetic relationships, gene duplication, conserved motifs, promoter cis-elements, and expression profiles under dark-induced leaf senescence. Based on transcriptome and RT-qPCR analyses, SiUGT26 was selected as a senescence-responsive candidate gene for preliminary functional analysis in Arabidopsis. These results provide a useful resource for the SiUGT gene family and candidate genes for future studies on UGT-mediated leaf senescence in foxtail millet.

## Materials and methods

### Retrieval of the foxtail millet UGT gene family database

The reference genome data for the “xiaomi” genome were obtained from the Multi-Omics Database for Setaria italica (Li et al., 2023). The Hidden Markov Model (HMM) corresponding to the UGT conserved domain (PF00201) was obtained from the Pfam and SMART databases (Letunic and Bork, 2018). The HMMER3.0 software was employed to identify the UGT structural domain within the parameter of an E-value of 10−10 or lower (Finn et al., 2015). A total of 122 Arabidopsis UGT protein sequences were selected as query sequences, and the BLASTP tool was used to compare these sequences with those of Arabidopsis, setting an E-value threshold of <1 × 10−10. After excluding incomplete PSPG-box proteins, 173 SiUGT protein sequences were identified. Additionally, 122 AtUGT protein sequences were retrieved from the CAZy database, and genes containing conserved UGT domains were selected for further analysis. The ExPASy online tool was used to analyze the isoelectric point (pI) and molecular weight (MW) of the UGT protein sequences from foxtail millet (Duvaud et al., 2012). Furthermore, Cello software was employed to predict the subcellular localization of gene families (Pierleoni et al., 2006).

We used the MEME online tool to analyze 173 conserved motifs in UGT protein sequences. The maximum number of patterns was set to 12, with other parameters set to default (Bailey et al., 2015). Additionally, the NCBI CDD tool was used to analyze the conserved structural domains in the 173 “xiaomi” UGT protein (Marchler-Bauer et al., 2015). Exons, introns, and non-coding regions (UTRs) corresponding to the coding sequences (CDSs) of the 173 UGT genes were extracted from the “xiaomi” genome data for gene structure analysis. CFVisual v2.1.5 was then used to extract, map, and visualize the UGT exon and intron information (Chen H. et al., 2022).

### Phylogenetic analysis of UGT genes in foxtail millet, Arabidopsis, and maize

MUSCLE was used to align the UGT protein sequences from foxtail millet, Arabidopsis, and maize. The resulting alignment was then trimmed using TBtools-II v2.096 to remove gaps (Chen et al., 2023). A phylogenetic tree was constructed for the UGT genes of foxtail millet, along with 14 AtUGTs from Arabidopsis and 3 UGTs from maize, using MEGA 7.0 (Kumar et al., 2016). The tree was generated using the proximity method, with 1000 bootstrap replicates for parameter validation. SiUGTs were classified according to the subgroups and branches in the phylogenetic tree based on previous studies (Li et al., 2014; Lescot et al., 2002). Finally, Adobe Illustrator 2023, version 27.0 was used to enhance the visualization.

### Chromosomal distribution, gene replication, and collinearity analysis

To determine the distribution of UGT genes on the foxtail millet chromosomes, we retrieved genomic location data and used TBtools to map the gene positions (Chen et al., 2023). TBtools was also employed to analyze the tandem and segmental repeats of SiUGT genes in foxtail millet. To determine the replication pattern of each SiUGT gene within the foxtail millet genome, we used MCScanX to assess genomic collinearity with default parameters. Additionally, MCScanX was employed for collinearity analysis between foxtail millet and other species, including Arabidopsis, rice, sorghum, and maize.

### Identification of Cis-regulatory elements in the promoter regions of SiUGT genes in foxtail millet

The 2000 bp DNA sequences of the upstream SiUGTs promoter regions from the foxtail millet genome were extracted using TBtools. These sequences were then submitted to the Plant CARE database, which identifies the functions of cis-elements and screens for stress responses, as well as factors involved in plant growth, development, and hormone regulation (Lescot et al., 2002; Gachon et al., 2005).

### RT-qPCR analysis

For regional expression profiling, light-, moderate-, and severe-senescence regions were sampled along senescing foxtail millet leaves. These categories were defined operationally by visible leaf-color progression together with position along the leaf blade and are distinct from the LS and SS variety designations used for the published transcriptome dataset. Three independent biological replicates were analyzed. Relative transcript abundance was calculated using the 2^−ΔΔCt^ method (Livak and Schmittgen, 2001) after normalization to the internal reference gene used in the experiment. Primer sequences are provided in **Supplementary Table S3**. The three-region experiment was used primarily for candidate prioritization; therefore, the expression profiles are presented descriptively as mean ± SD without pairwise significance claims based on repeated Student’s t-tests.

### Co-expression analysis of SiUGT and SiWRKY genes

Co-expression analysis was performed using the published DIS transcriptome expression matrix. Seven expressed group E SiUGT genes and 21 expressed SiWRKY genes were included in the exploratory screen, and pairwise Pearson correlation coefficients were calculated to identify expression associations. The analysis was used to prioritize co-expression candidates rather than to infer direct transcriptional regulation. Because multiple UGT–WRKY pairs were screened, the correlation coefficient alone was not interpreted as evidence that a WRKY gene is an upstream regulator of a *SiUGT* gene.

### Expression patterns of *SiUGT* genes

The foxtail millet genome served as a reference for sequence alignment and subsequent analysis. Transcriptome data of dark-induced leaf senescence, expressed as FPKM, from our research group were used to investigate the expression patterns of *SiUGT*s. Clean read sequences were aligned with the reference genome using STAR, a specialized RNA-seq alignment software, to determine the locations of reads on the genome or within specific genes. STAR aligned the sequenced transcriptome reads to the genome, identified splicing connections between exons, and compared a larger number of reads to the reference genome, thus enhancing the utilization of sequencing data. Reads aligned to the reference genome are called mapping reads, and the subsequent analysis was based on these mapped reads.

### Subcellular localization of SiUGT26

The coding sequence (CDS) of the *SiUGT26* gene (1413 bp) was PCR-amplified using “*xiaomi*” cDNA as the template. *Hind* III and *Nco* I cleavage sites were added to the upper and lower primers, respectively, and the amplified product was inserted into the pCAMBIA1300 binary expression vector via *Hind* III/*Nco* I double digestion. The resulting plant expression vector, carrying the *35S-SiUGT26* construct, was transformed into *Escherichia coli* strain DH5α. The cloned plasmid was extracted and transformed into *Agrobacterium* strain GV3101. The foxtail millet protoplasts were then transformed and incubated in the dark. Finally, the fluorescence signal of the green fluorescent protein (GFP) was observed using an Olympus confocal microscope(Evident Corporation (Olympus Life Science), Tokyo, Japan).

### Construction of an overexpression vector and transformation of Arabidopsis

The recombinant plant expression vector 35S-SiUGT26 was introduced into Arabidopsis via the flower dip method (Bent, 2006). Seeds of 35S-SiUGT26 were screened on 1/2 MS medium with 25 mg/L hygromycin, yielding six homozygous lines. Total RNA was extracted from the T3 generation homozygous transgenic plants, reverse-transcribed into cDNA, and SiUGT26 expression was quantified by RT-qPCR. Subsequently, three high-expression representative lines were selected for further experiments.

### Plant materials

The Stage 6.00 Arabidopsis plant (Boyes et al., 2001) was collected. The 5-week-old foxtail millet top second leaf was used for subsequent quantitative experiments. All samples were immediately immersed in liquid nitrogen and stored at −80 °C for further analysis. Gene expression levels were calculated using the 2−ΔΔCt method (Livak and Schmittgen, 2001).

## Results

### Identification of *SiUGT* genes in foxtail millet

To identify candidate genes of the UGT family in foxtail millet, we used the HMM profile of the UGT conserved domain (PF00201) and 122 *Arabidopsis UGT* genes as queries. A total of 173 *UGT* members were identified in the genome, designated *SiUGT001-SiUGT173* according to their chromosomal positions. The protein sequences encoded by *SiUGT* genes ranged in length from 167 amino acids (*SiUGT86*) to 948 amino acids (*SiUGT54*), with an average of 482 amino acids. Notably, 47.98% of SiUGT proteins exceeded 482 amino acids in length. The relative molecular weights (MW) varied from 17.71 kDa (*SiUGT86*) to 101.41 kDa (*SiUGT54*), with an average molecular weight of 52.03 kDa. The theoretical isoelectric point (pI) values ranged from 4.88 (*SiUGT32*) to 11.58 (*SiUGT66*), with an average pI of 5.74. The prediction of subcellular localization revealed that 70 of the proteins were distributed between the cytoplasm and chloroplasts, 18 were associated with the plasma membrane, 14 were localized in mitochondria, and one was found in the nucleus. The detailed physicochemical properties of all *SiUGT* genes, including amino acid length, MW, pI, and subcellular localization, were provided in **Supplementary Table S1**. The results indicated that the molecular weight, isoelectric point, and predicted subcellular localization of these proteins varied widely, suggesting potential functional diversity within the UGT family in foxtail millet.

### Chromosomal distribution of the SiUGT gene family

To investigate the chromosomal locations of *SiUGT*s, we constructed a chromosome map to determine the distribution of *SiUGT* protein-coding genes in foxtail millet. As shown in **Figure 1**, 169 *SiUGT*s were unevenly distributed across the nine chromosomes, with gene counts ranging from four to 36 per chromosome. The remaining four *SiUGT*s were located on unassembled contigs within the genome. Chromosome 2 contained the highest number of *SiUGT*s (36, *SiUGT24-SiUGT59*), representing 20.81% of the total gene population. Chromosome 9 harbored 27 genes (*SiUGT143-SiUGT169*), accounting for 15.61%. In contrast, chromosomes 6 and 8 contained the fewest *SiUGT* genes, each with seven genes (*SiUGT105-SiUGT111* and *SiUGT136-SiUGT142*), together representing 4.05%. A ranking of UGT family members based on their chromosomal and scaffold locations reveals a clustering pattern of *SiUGT*s across the chromosomes.

**Figure 1:**
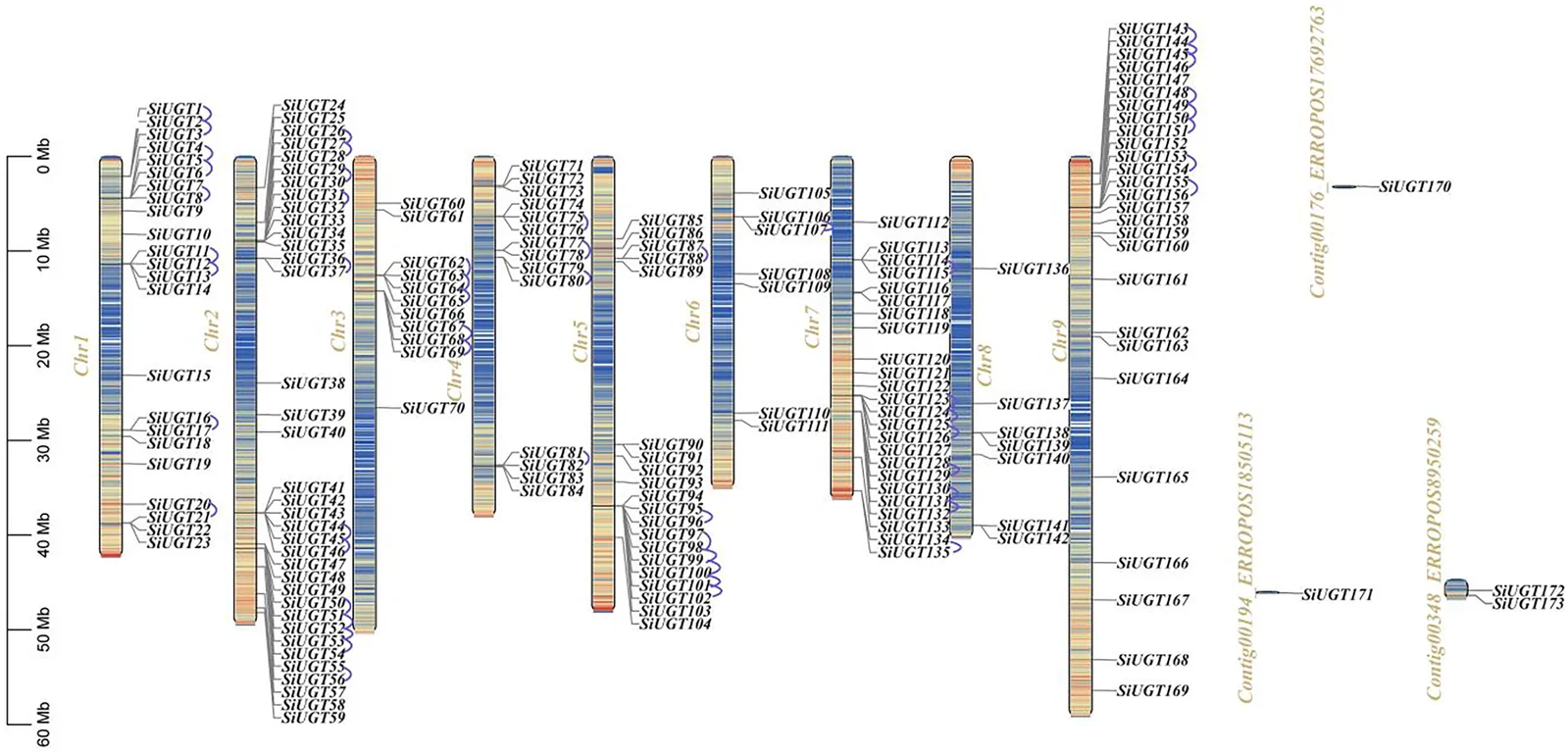
Chromosome mapping of UGT genes in foxtail millet. The line on the gray strips shows the location of SiUGTs on chromosomes. The scale on the left of the picture is in megabases (Mb). Tandem duplications are indicated by blue lines.

### Phylogenetic analysis of UGTs in foxtail millet, Arabidopsis, and maize

Because UGT proteins clustered within the same subgroup may share related evolutionary origins and potential functional similarities, we compared the identified SiUGT proteins with representative UGT members from Arabidopsis and maize. This analysis was used to classify SiUGTs into conserved UGT subfamilies and to clarify their phylogenetic relationship with Arabidopsis UGTs. Phylogenetic analysis of 173 identified SiUGT proteins indicates that they can be classified into 17 distinct subfamilies, including three newly identified groups (O, P, and Q) in both *Arabidopsis* (Li et al., 2001) and maize (Li et al., 2014). Because SiUGT26 was further examined in a heterologous Arabidopsis system, we paid particular attention to the relationship between SiUGTs and Arabidopsis UGT members. In the phylogenetic tree, Arabidopsis UGTs served as reference sequences for subgroup classification. SiUGT26 was assigned to subfamily E, which also contained several Arabidopsis UGT members. This result suggests that SiUGT26 shares a certain degree of evolutionary conservation with Arabidopsis UGT proteins, although a direct one-to-one orthologous relationship was not established in this analysis. The phylogenetic tree of these proteins further confirms this classification (**Figure 2**). Among the 17 subfamilies, subfamily E contains the highest number of SiUGT proteins, with 43 members, followed by subfamily L, which includes 24 proteins. The total number of genes in the five subfamilies—E, L, D, G, and H—represents 70% of the overall gene count. In contrast, subfamilies J and K have the fewest SiUGT proteins, with only one member each. Notably, the three newly identified subfamilies show a distinct clustering pattern in foxtail millet compared to other species. UGT sequences with similar structural features tend to cluster in the same branch of the phylogenetic tree, and UGT proteins within the same subfamily often share a common evolutionary lineage. This provides valuable insights for future research on UGT protein functions. These results indicate that Arabidopsis UGTs provide useful phylogenetic references for SiUGT classification, while the function of SiUGT26 in Arabidopsis should be interpreted as heterologous functional evidence.

**Figure 2:**
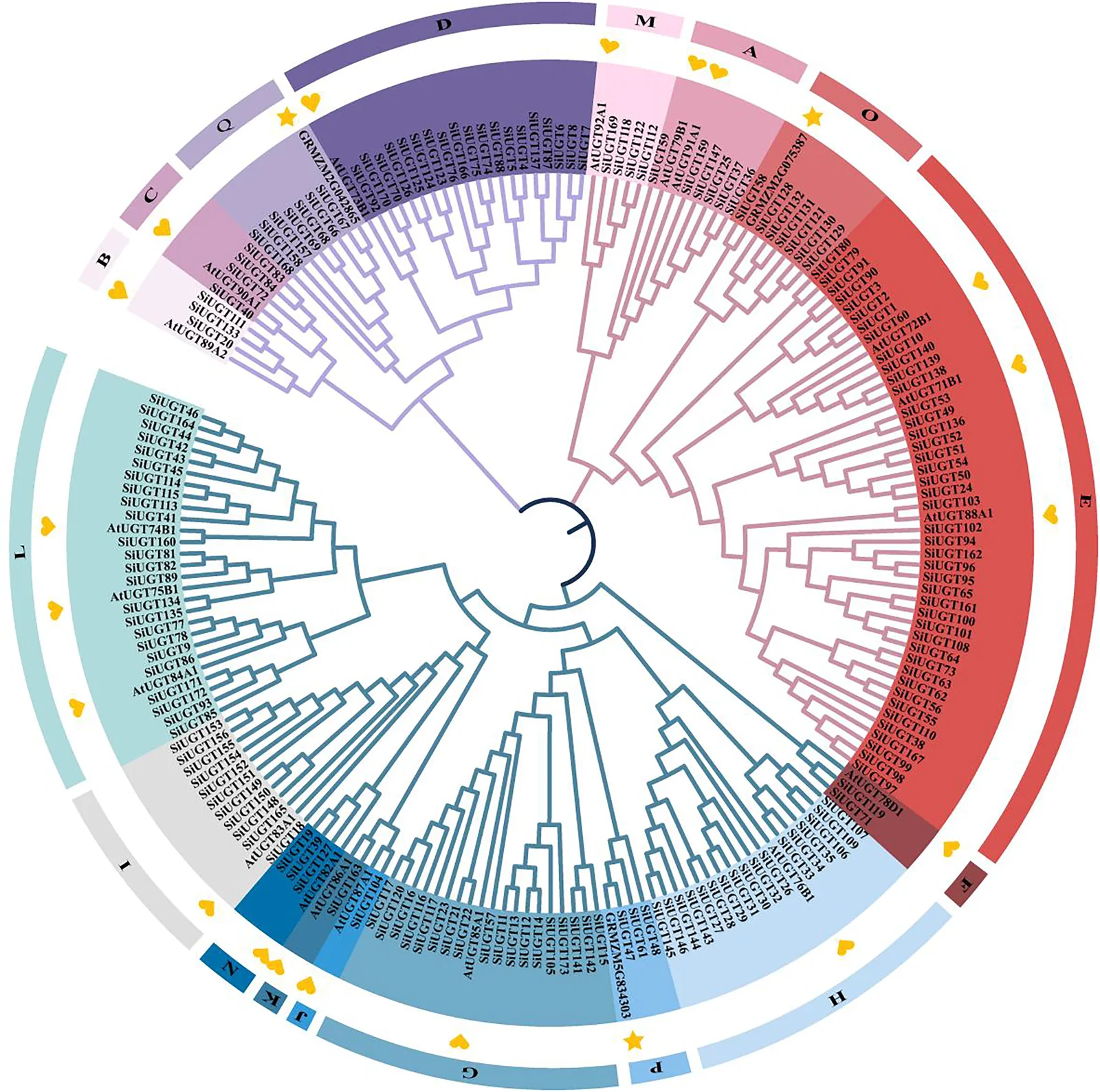
Neighbor-joining phylogenetic tree constructed with the UGT proteins incorporating a total of 173 UGT proteins from foxtail millet, 19 UGT proteins from Arabidopsis (♥) and 3 UGT proteins from maize (☆). The resulting proteins were categorized into 17 distinct subgroups. The representation of each subgroup is differentiated by unique colors and graphics, labeled **(A–Q)**.

### Analysis of conserved motifs, domains, and gene structure of *SiUGTs*

To further explore the characteristics of *SiUGT*s, we analyzed the conserved motifs and gene structures of *SiUGT*s using the phylogenetic tree (**Figure 3A**). A total of 12 conserved motifs with 11 to 50 amino acids were identified in 173 SiUGT proteins. These motifs ranged in length from 11 to 37 amino acids. Each SiUGT protein contained 4 to 12 motifs, with the majority concentrated in the N-terminal region (**Figure 3B**). Among them, most of the SiUGT members contained more than 4 conserved motifs, except SiUGT85 and SiUGT86. Motifs 1, 3, and 11 were commonly found in SiUGT members, with motif 3 present in all SiUGTs. Motif 1 was found in 172 SiUGT members, excluding SiUGT25, whereas motif 11 was identified in 171 members, with SiUGT136 and SiUGT159 as exceptions. To better understand the role of intron-exon structures in the evolution of polygenic families, we visualized genome annotation data related to these structures. Among the 173 subfamily members, 90 *UGT* genes (52%) were intronless, whereas 1 exon was present in 52% of the genes, 2 exons in 33.5%, 3 exons in 10% (10 members), 4 exons in 2.8% (5 members), and only one exon was identified in *SiUGT159*. We analyzed the amino acid sequences of 173 SiUGT proteins using the Conserved Domain Database (CDD) and identified four conserved domains (**Figure 3C**). The analysis of gene structure and motifs suggests that the arrangement of gene sequences and conserved protein motifs within subfamily members may be linked to their functional conservation, potentially due to structural variations that have occurred during evolution.

**Figure 3:**
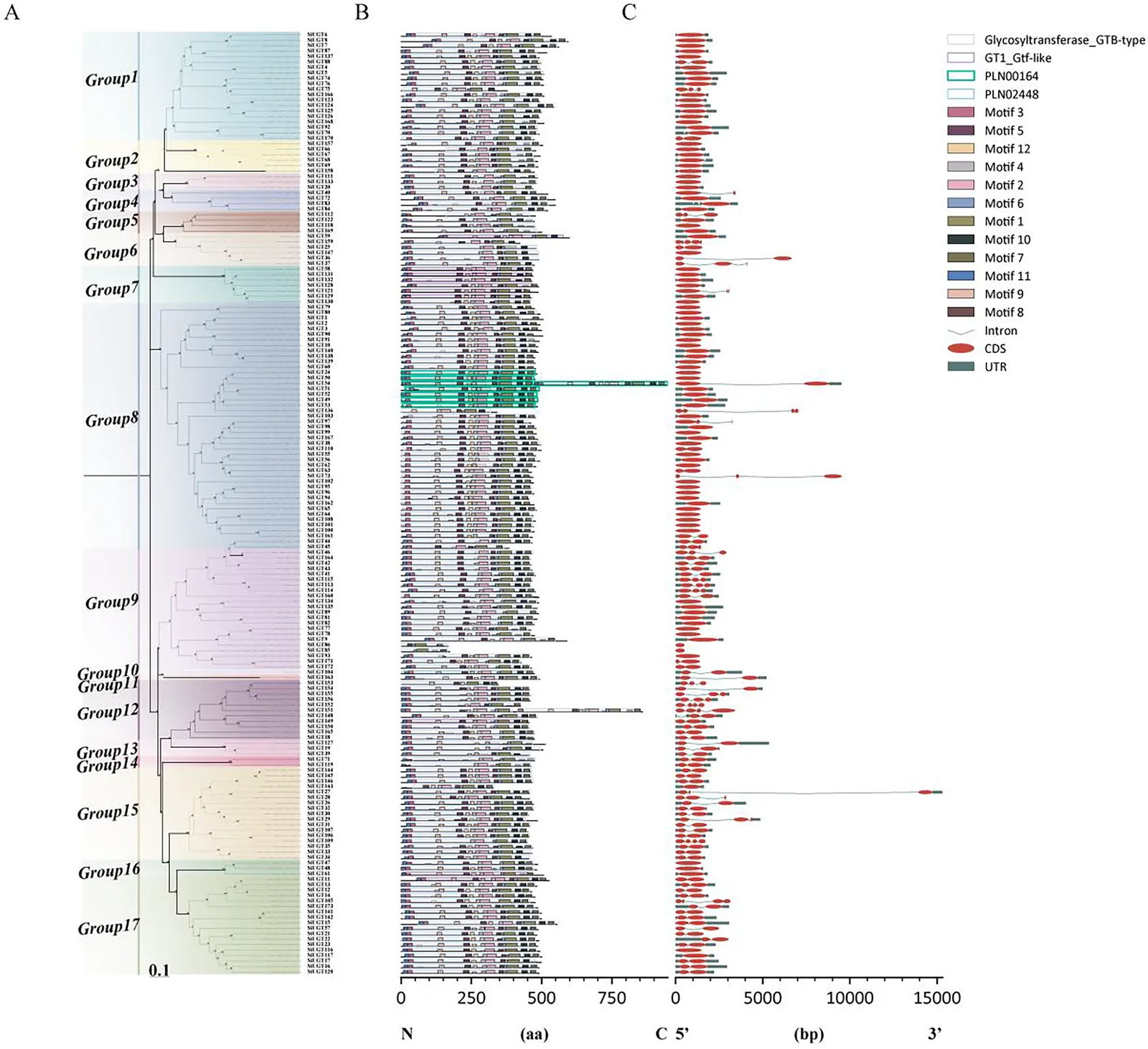
**(A)** Phylogenetic tree of UGT gene family members. **(B)** MeMe motifs and conserved domains. **(C)** Gene structures in foxtail millet. The red ovals represent exons, the black lines represent the introns, and the green boxes represent upstream/downstream sequences.

### Gene duplication and collinearity among *SiUGT* genes in foxtail millet

Gene duplication plays a key role in the formation, expansion, and functional diversification of gene families. Gene duplication analysis of 173 *SiUGT*s identified 79 pairs (91.33%) of tandem repeats, distributed across all eight chromosomes of the foxtail millet genome (**Figure 4**). Specifically, the duplicated genes were distributed across chromosome 1 (9 clusters), chromosome 2 (12 clusters), chromosome 3 (5 clusters), chromosome 4 (4 clusters), chromosome 5 (7 clusters), chromosome 6 (1 cluster), chromosome 7 (8 clusters), and chromosome 9 (8 clusters), with no tandem genes on chromosome 8. These 79 pairs of duplicated genes exhibit a high degree of homology, suggesting that some *SiUGT* genes may have originated from gene duplication. A total of 10 duplicate gene pairs were identified among the 173 *SiUGT* genes, including *SiUGT4*/*SiUGT87*, *SiUGT19*/*SiUGT127*, *SiUGT20*/*SiUGT111*, *SiUGT29*/*SiUGT144*, *SiUGT62*/*SiUGT96*, *SiUGT63*/*SiUGT97*, *SiUGT81*/*SiUGT89*, *SiUGT15*/*SiUGT120*, *SiUGT122*/*SiUGT173*, and *SiUGT24*/*SiUGT147*. These findings suggest that the chromosomal fragments likely originated from a replication event during evolution. The genes have not fully differentiated, which may result in functional redundancy. Ka/Ks represents the ratio of the non-synonymous replacement rate (Ka) to the synonymous replacement rate (Ks) between two protein-coding genes. This ratio indicates whether selection pressure is acting on the gene. The Ka/Ks value (Zhang, 2022) of ten *SiUGT* gene pairs was <1, indicating that the *UGT* gene family in foxtail millet has undergone purifying selection throughout its evolutionary history (**Supplementary Table S2**). Therefore, tandem repeats and large fragment duplications play a crucial role in the expansion of the *SiUGT* gene family and may serve as primary drivers of *SiUGT* gene evolution. The identification of multiple segment repeats and tandem repeats indicates the presence of interfering genes at adjacent loci during the expansion of the *SiUGT* gene family.

**Figure 4:**
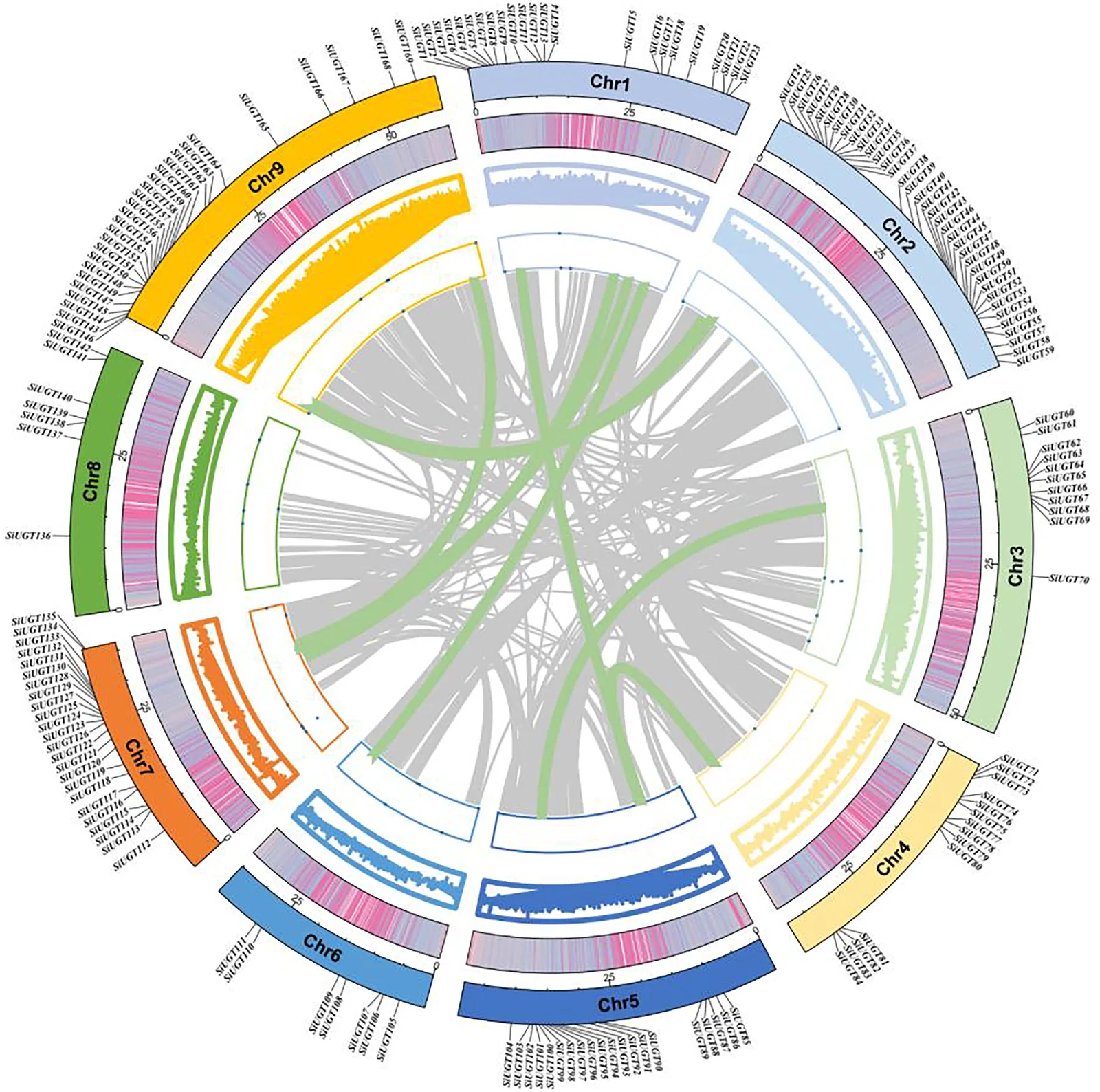
Segmental replication events of SiUGT genes. The gray lines indicate all the same linear blocks in the foxtail millet genome, and the green lines indicate the segmental repeats of UGT genes in foxtail millet.

To further explore the evolutionary relationships of *SiUGTs*, we conducted a comparative homology analysis between foxtail millet and dicotyledonous plants (*Arabidopsis*) as well as monocotyledonous plants (rice, maize, and sorghum). Comparative collinearity analysis was performed between foxtail millet and Arabidopsis, rice, sorghum, and maize. Among the 173 *SiUGT* genes, collinear relationships were detected with 2 Arabidopsis genes, 73 sorghum genes, 66 rice genes, and 61 maize genes, respectively (**Figure 5**). The number of collinear *UGT* gene pairs between foxtail millet and Arabidopsis was much lower than that between foxtail millet and the monocotyledonous species. This result indicates that *SiUGTs* are evolutionarily more closely related to *UGTs* from monocotyledonous crops, whereas their collinearity with Arabidopsis *UGTs* is relatively limited. Therefore, Arabidopsis was used in this study mainly as a heterologous system for preliminary functional validation of *SiUGT26*.

**Figure 5:**
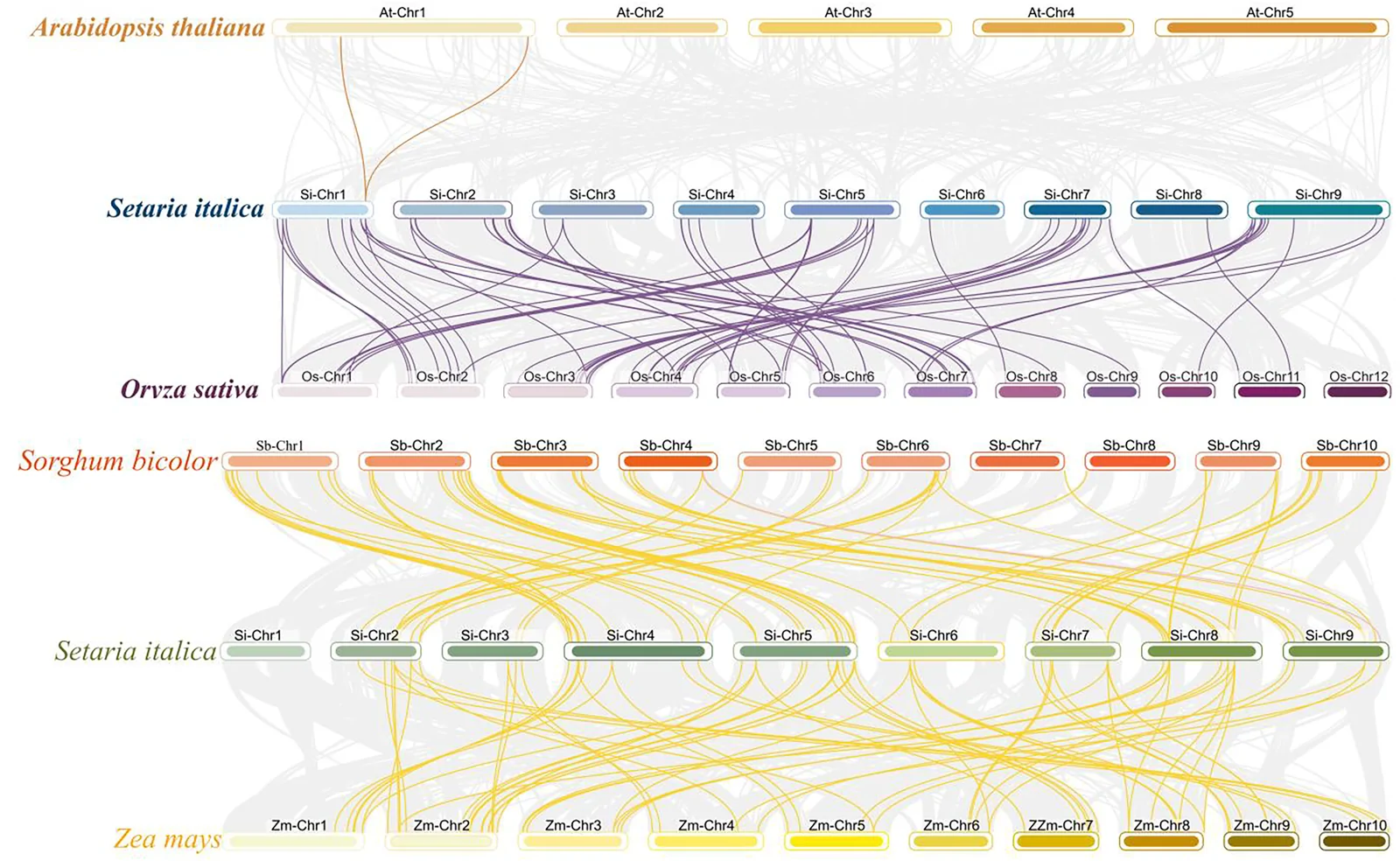
Collinearity analysis of UGT genes in Setaria italica, Arabidopsis thaliana, Oryza sativa, Sorghum bicolor and Zea mays.

### Analysis of Cis-acting elements and expression patterns of SiUGT genes

To investigate the potential transcriptional regulatory mechanisms of *SiUGT* genes, we analyzed the cis-acting elements in their promoter region. This study identified cis-regulatory elements in three functional categories: hormone response, plant growth and development, and stress response (**Figure 6**). Stress-responsive elements, including low temperature (LTR), drought (MBS), and defense/stress response (TC-rich repeats), as well as hormone-responsive elements such as salicylic acid (TCA-element), jasmonic acid (TGACG-motif, CGTCA-motif), gibberellin (GARE-motif, TATC-box, P-box), abscisic acid (ABRE), and auxin (TGA-element, AuxRR-core), were identified. Additionally, growth regulation-related elements, including hypoxia-specific induction (GC-motif), meristem-specific expression (CAT-box), and MYBHv1 binding sites (CCAAT-box), were also observed. This classification suggests that *SiUGT* genes are likely regulated by various cis-acting elements in their promoter regions, which influence their expression during foxtail millet growth and development, as well as in response to stress. The presence of multiple cis-acting elements in gene promoter regions is essential for enabling responses to various stress conditions, indicating that these elements play specific roles in stress regulatory networks. Furthermore, *SiUGT* genes belonging to the same category demonstrate a range of types and quantities of stress and hormone response elements, indicating a nuanced and complex framework of expression and regulation among different *SiUGT* genes.

**Figure 6:**
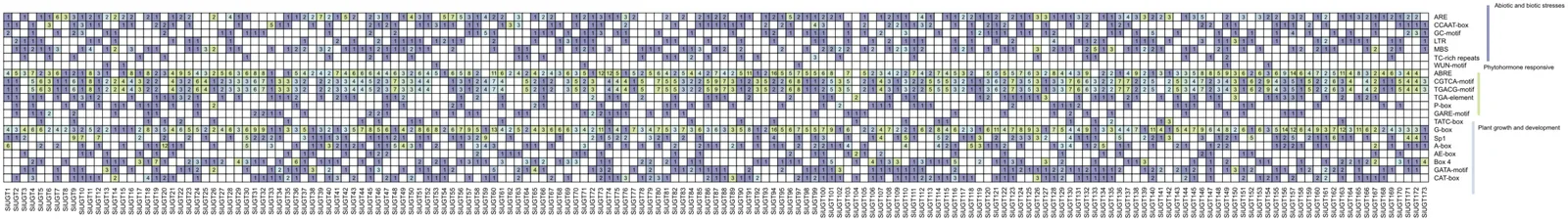
Analysis of cis-acting regulatory elements in SiUGT promoters. The depth of color in the image indicates the quantity; the lighter colors represent a small amount or none, whereas the darker colors represent a large amount.

The analysis of cis-acting elements suggests that the *SiUGT* genes may regulate foxtail millet development and stress responses. Senescence is a key process in plant development, essential for promoting phenotypic plasticity and enabling adaptation to environmental stressors. Therefore, we further analyzed the expression patterns of *SiUGT* genes associated with leaf senescence using transcriptome data from dark-induced senescence (DIS) in foxtail millet (Zhen et al., 2024). We found that 48 *SiUGT* genes were down-regulated and 7 were up-regulated in the light senescence (LS) variety during DIS, whereas 38 were up-regulated and 15 genes were down-regulated in the severe senescence (SS) variety (**Figure 7**). To further investigate the expression patterns of *SiUGT* genes in different regions of senescent foxtail millet leaves, 12 genes were analyzed using RT-qPCR. The results indicated that two *SiUGT* genes were significantly down-regulated in the severely senescent region, particularly *SiUGT26*, which was barely expressed in this region. In contrast, *SiUGT62* was highly expressed in moderately senescent regions. In the severely senescent region, the expression levels of all selected *SiUGT* genes did not show significant differences (**Figure 8**). These data suggest that *SiUGT26* and *SiUGT62* may have distinct roles in regulating leaf senescence in foxtail millet.

**Figure 7:**
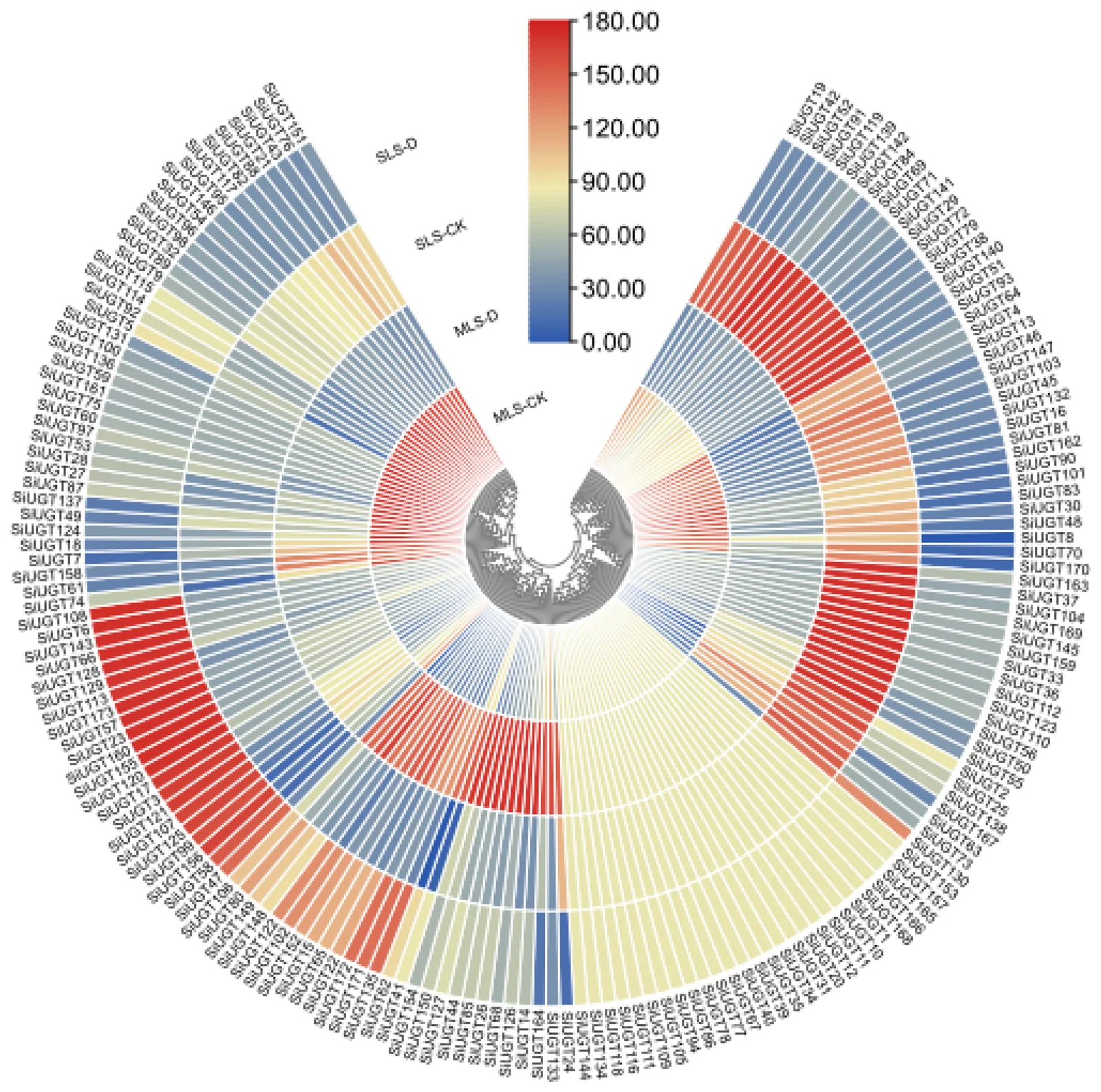
The expression analysis of SiUGTs under dark-induced leaf senescence. MLS represents light senescence samples, and SLS represents the severe senescence samples.

**Figure 8:**
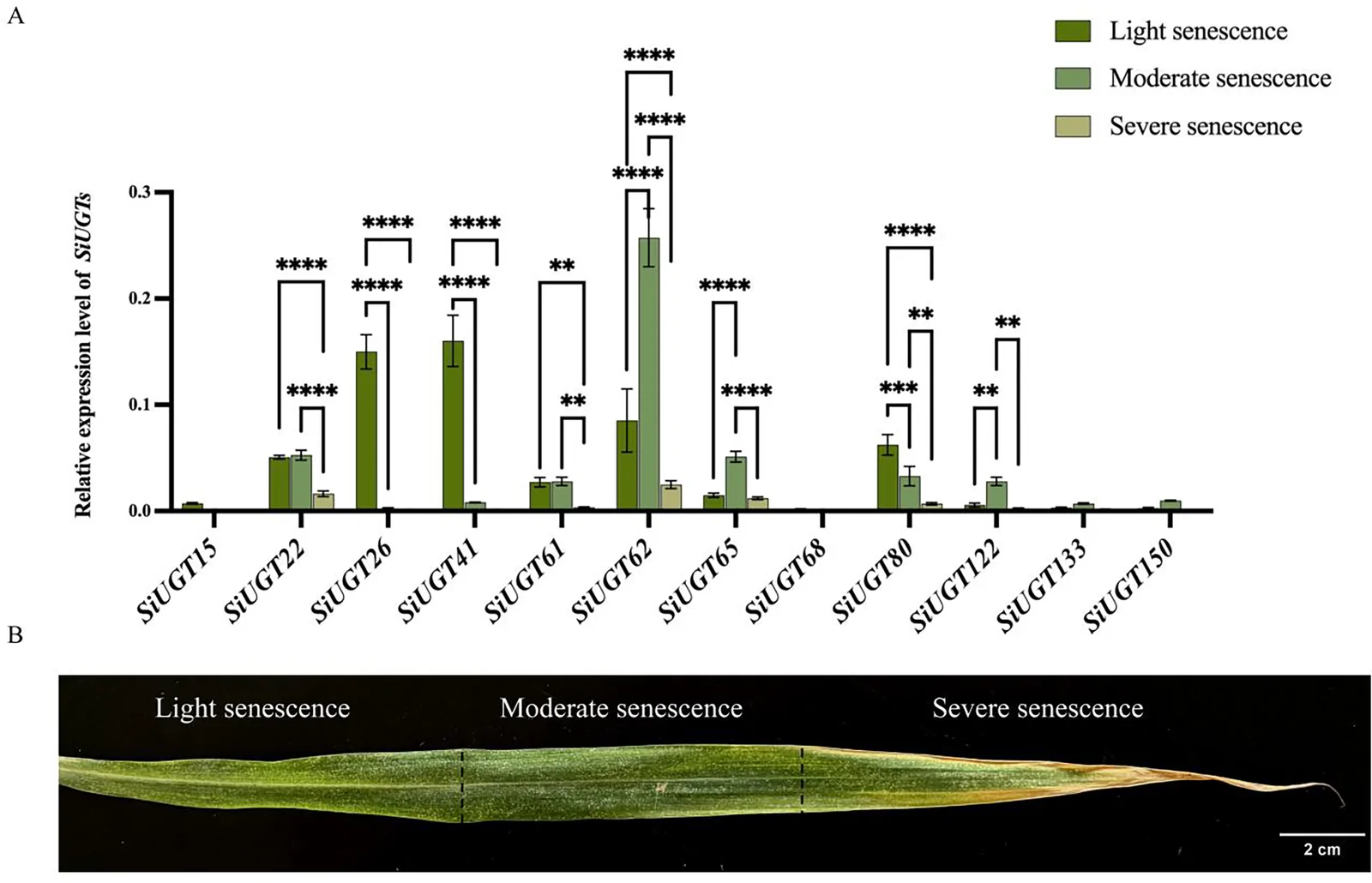
Relative expression levels of 12 SiUGTs genes in foxtail millet at different regions of leaf senescence. **(A)** Expression levels of 12 SiUGTs genes in foxtail millet at different senescence stages. All data are presented as means ± SD from three independent replicates. **P < 0.01, ***P < 0.001, and ****p < 0.0001. *p*-values were determined by Student’s *t*-test. **(B)** Photograph of foxtail millet leaves at different senescence stages. Scale bar: 2 cm.

### Heterologous overexpression of SiUGT26 was associated with growth and senescence-related phenotypes in Arabidopsis

To obtain preliminary evidence for the potential function of SiUGT26, transgenic Arabidopsis lines constitutively expressing SiUGT26 were evaluated, and three representative homozygous lines (OE-3#, OE-5#, and OE-8#) were selected for phenotypic characterization (**Figures 9A, B**). Compared with wild-type Col-0, the SiUGT26-overexpressing lines showed larger rosettes (**Figures 9C, D**). The transgenic lines also showed higher Fv/Fm values (**Figure 9E**) and lower transcript levels of the selected senescence-associated marker genes AtSAG12, AtSAG13, and AtSAG113 (**Figures 9F–H**). These observations indicate that heterologous expression of SiUGT26 was associated with altered rosette growth and senescence-related physiological and molecular traits in Arabidopsis. Because the representative rosette phenotype does not independently establish delayed senescence and the assay was performed in a heterologous system, these results are interpreted as preliminary evidence and do not establish the native function of SiUGT26 in foxtail millet. Subcellular localization analysis showed that the SiUGT26–GFP signal was detected in both the nucleus and cytoplasm (**Figure 10**). Because no nuclear-marker co-localization assay was performed, preferential nuclear localization was not inferred.

**Figure 9:**
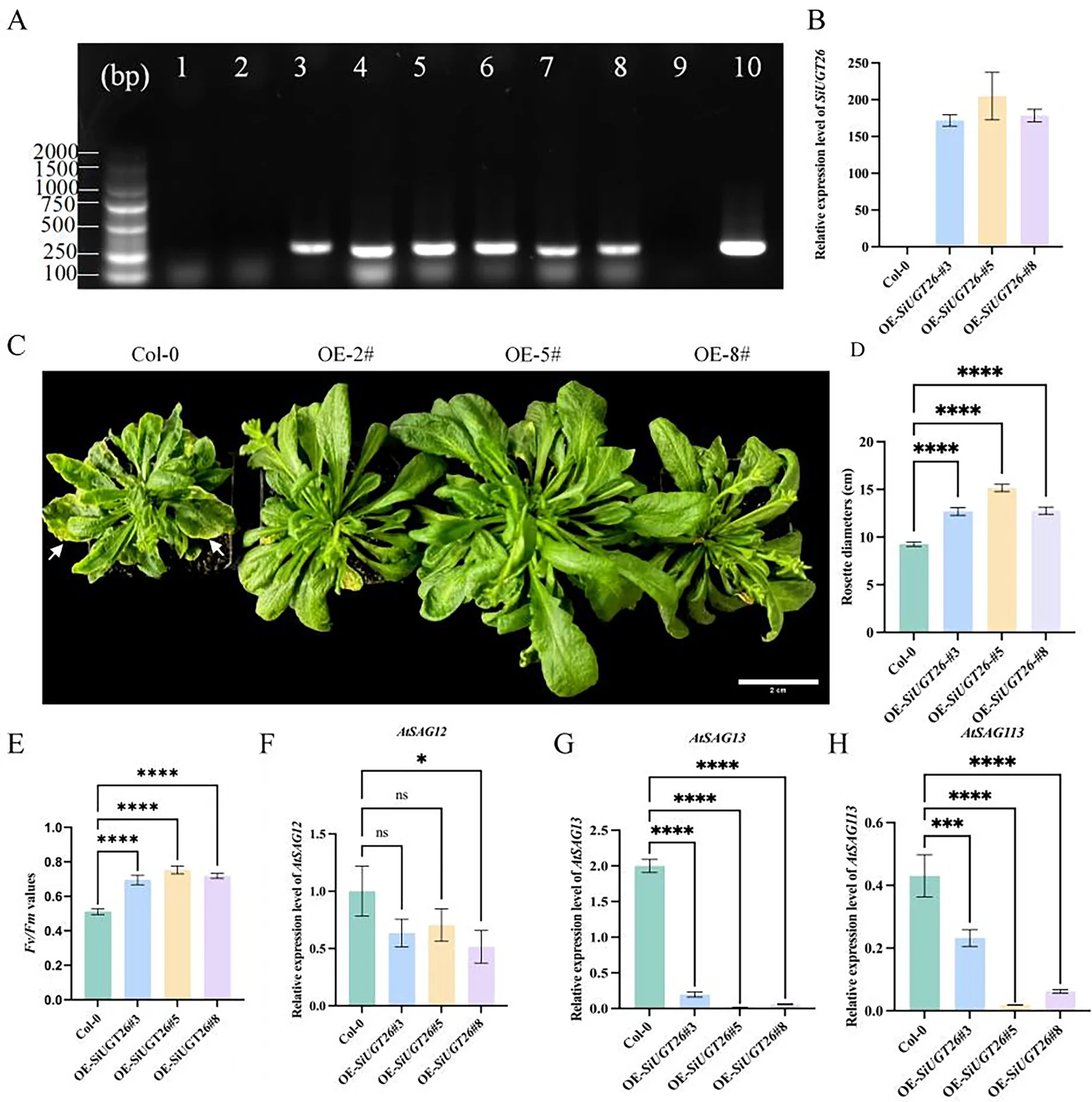
Phenotypic characterization of SiUGT26-overexpressing Arabidopsis lines during senescence. **(A)** PCR validation of SiUGT26 transgene integration in homozygous lines (1–8). M: 2000 bp DNA marker; -: wild-type negative control (9); +: positive plasmid control (10). **(B)** RT-qPCR analysis of SiUGT26 transcript levels in transgenic lines. **(C)** Representative rosette phenotypes in wild type and SiUGT26 transgenic lines. **(D)** Quantification of rosette diameters, **(E)** maximum quantum yield of photosystem II (Fv/Fm). (**F**–**H**) Relative expression of senescence-associated marker genes (AtSAG12, AtSAG13, AtSAG113). Data represent mean ± SD (n = 3 biological replicates). Statistical significance: ns: no significant difference, *p < 0.05, ***P < 0.001, and ****p < 0.0001 (Student’s *t*-test). Scale bar: 2 cm.

**Figure 10:**
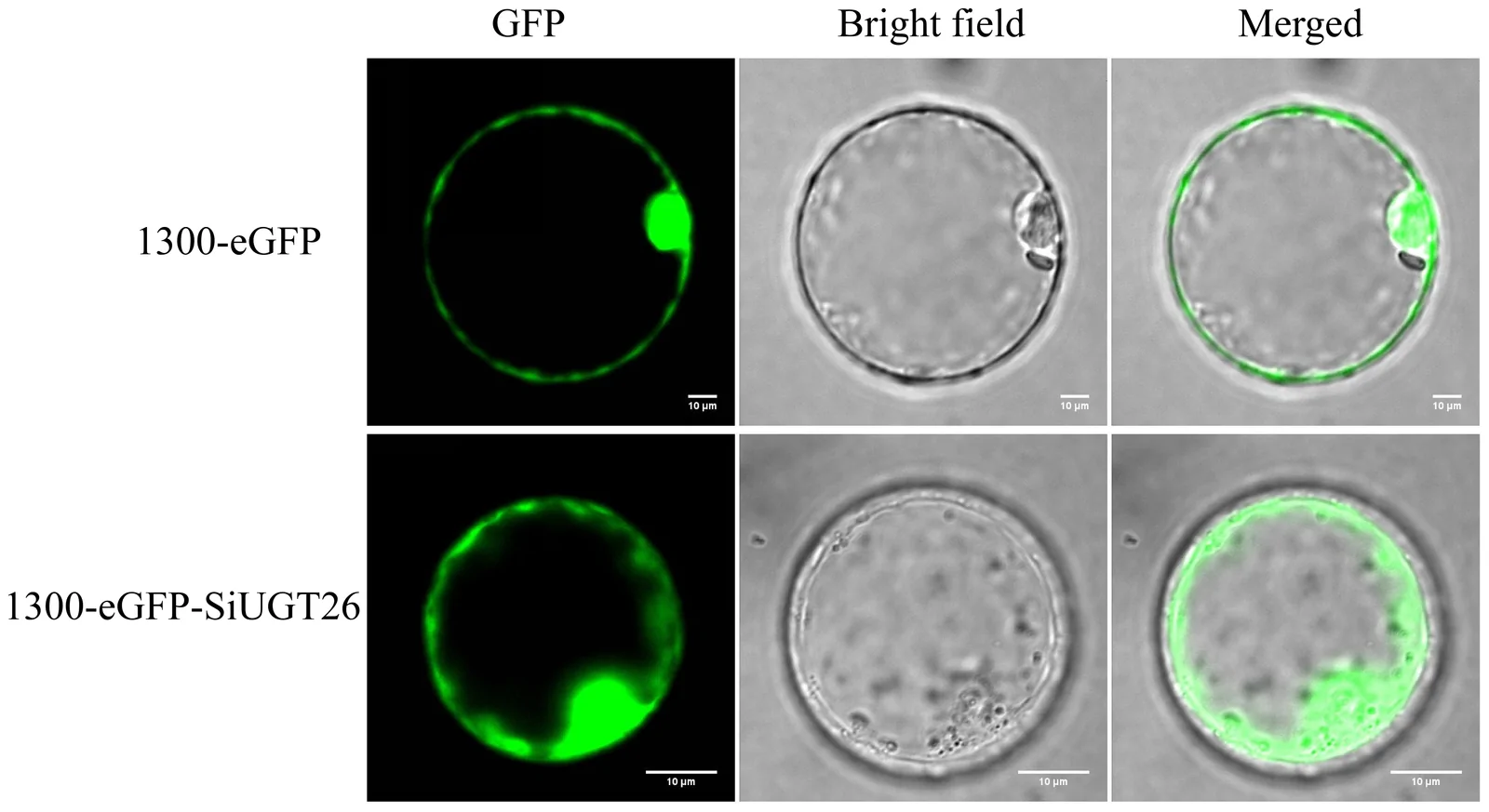
Subcellular localization of SiUGT26 in foxtail millet protoplasts. The SiUGT26–GFP fluorescence signal was detected in the nucleus and cytoplasm. Because a nuclear-marker co-localization assay was not performed, preferential nuclear localization is not inferred.

### Co-expression analysis identified SiWRKY71 as a candidate associated with SiUGT26 expression

Since WRKY transcription factors play a crucial role in regulating leaf senescence (Jiang et al., 2017)To explore candidate transcription factors associated with SiUGT26 expression, expression correlations were examined between seven expressed group E SiUGT genes and 21 SiWRKY genes using the published DIS transcriptome dataset. SiUGT26 and SiWRKY71 showed a positive expression correlation (r = 0.719; **Figure 11**). This result identifies SiWRKY71 as a co-expression candidate associated with SiUGT26 expression during dark-induced leaf senescence. Because multiple UGT–WRKY pairs were screened and correlation does not demonstrate promoter binding or transcriptional activation, the present analysis does not establish SiWRKY71 as an upstream regulator of SiUGT26. Direct regulation will require independent experimental validation.

**Figure 11:**
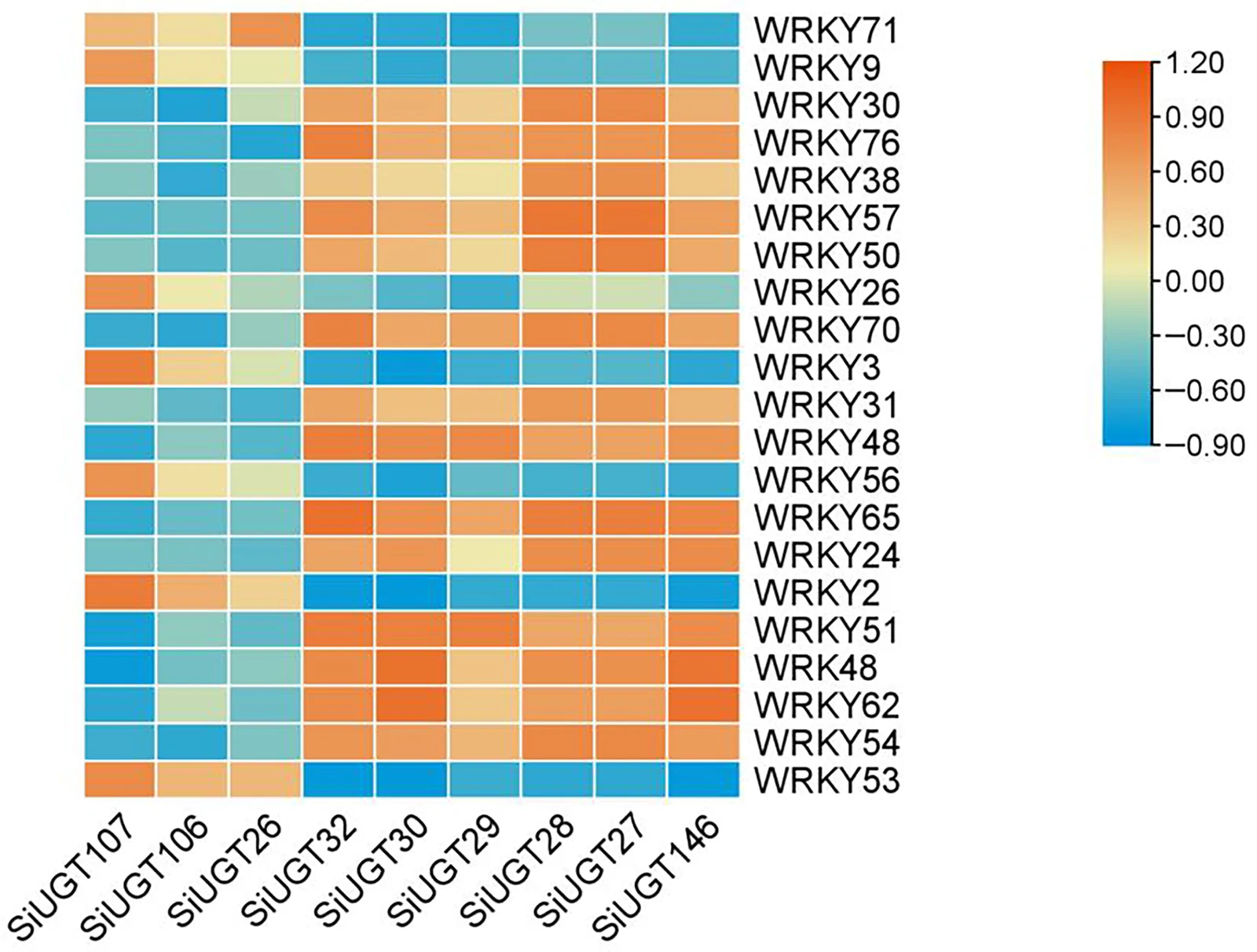
Pearson correlation coefficients between seven expressed group E SiUGT genes and 21 SiWRKY genes in the published dark-induced leaf senescence transcriptome dataset. Correlations are used to identify co-expression candidates and are not interpreted as evidence of direct transcriptional regulation.

## Discussion

Glycosylation mediated by UDP-glycosyltransferases (UGTs) is an important enzymatic modification involved in plant secondary metabolism, abiotic and biotic stress responses, and developmental regulation (Wang, 2009). Although UGT families have been characterized in several plant species, their genomic organization and senescence-associated expression in foxtail millet remain incompletely understood. Our genome-wide analysis identified 173 SiUGT genes and provides a resource for comparative analysis in foxtail millet. Previous studies have reported extensive UGT gene-family organization and duplication in other plant species, including cotton and Arabidopsis (Huang et al., 2015; Galán-Vásquez and Perez-Rueda, 2019). Accordingly, the chromosomal clustering and duplication patterns observed here are interpreted primarily as features of SiUGT family organization rather than as evidence of specific regulatory interactions. Adjacent genes often share functional similarities or participate in the same metabolic pathways (Rehman et al., 2018), suggesting potential synergistic regulatory interactions. This clustering provides valuable molecular markers and insights for the genetic analysis of *SiUGT* genes in foxtail millet.

The classification of SiUGTs into 17 groups is consistent with previously established UGT phylogenetic frameworks in Arabidopsis and maize (Li et al., 2001, 2014). Groups O, P, and Q are therefore treated as previously defined UGT groups rather than novel subfamilies. SiUGT26 was assigned to group E together with several Arabidopsis reference UGT proteins, supporting subgroup-level evolutionary relatedness; however, the present NJ tree does not establish a one-to-one orthologous relationship or lineage-specific expansion. Group E was numerically the largest SiUGT group, followed by group L. Motif and gene-structure analyses further showed that members within several groups share similar structural features, while variation is also present among individual genes. These patterns are useful for family classification and candidate selection but do not, by themselves, establish gene function Discrepancies in conserved motifs were observed within the same subfamily, with different members exhibiting distinct motifs. This variability may contribute to the diverse and complex functions of *SiUGT*s, as Motifs 1, 3, and 5 correspond to the PSPG-box region. The constructed gene structure map indicated that members within the same subgroup share similar exon/intron counts, while substantial variations exist between different groups, suggesting an evolutionary divergence. Similar findings have been reported in soybean and buckwheat, indicating changes in intron number and location during the evolution of glycosyltransferase families, with consistent relative positions preserved within shared clades (Mamoon Rehman et al., 2016; Roy and Penny, 2007). Furthermore, variations in intron sizes across distinct evolutionary branches may arise from factors such as replication events, gene recombination, deletions, and loss of splicing sites during the evolution of the gene family.

Comparative collinearity analysis identified substantially more SiUGT relationships with the monocotyledonous species sorghum, rice, and maize than with Arabidopsis, which is consistent with their closer taxonomic relationships. The 10 segmentally duplicated SiUGT gene pairs analyzed in this study all had Ka/Ks values below 1, consistent with purifying selection. Promoter analysis also identified multiple predicted stress- and hormone-responsive cis-elements. These in silico predictions indicate potential regulatory complexity but do not demonstrate that the corresponding signaling pathways regulate individual SiUGT genes. It should also be noted that dark-induced leaf senescence is an experimental senescence model and may partially overlap with dark-stress responses. Therefore, DIS-responsive expression alone should not be interpreted as definitive evidence for a general role in developmental senescence. In the present study, the published DIS transcriptome dataset was used mainly to prioritize SiUGT genes associated with senescence-related responses.

Leaf senescence has been extensively investigated in model plants and major crops, particularly Arabidopsis and rice (Thomas, 2013; Guo et al., 2021). In comparison, the molecular regulation of leaf senescence in foxtail millet remains less well characterized, although recent transcriptomic work has begun to provide relevant candidate genes and regulatory information (Zhen et al., 2024). Previous work in cotton showed that UGT family members are associated with leaf-senescence responses and that silencing GHUGT116 can alter dark-induced senescence (Chen Y. et al., 2022). Current research predominantly focuses on the regulatory aspects of leaf senescence in rice and *Arabidopsis*; relatively limited attention has been given to the senescence processes in foxtail millet. Glycosylation, catalyzed by glycosytransferases, is a crucial modification essential for cellular growth, development, and metabolic balance (Yang et al., 2023). This process supports the diversity of small molecular compounds that play vital roles in seed germination, growth, and reproduction, including flowering and fruiting (Ren et al., 2022; Bauer et al., 2021). However, studies on the effects of glycosyltransferases on leaf senescence are limited. Previous studies in cotton have shown that several *GHUGT* genes, containing cis-acting elements related to ABA and JA, are involved in the regulation of leaf senescence. Chen et al. analyzed the expression profiles of *GHUGT*s and confirmed the role of GHUGT116 in delaying dark-induced leaf senescence (Chen Y. et al., 2022). The glucosyltransferase AtUGT76B1 is closely associated with senescent phenotypes in *Arabidopsis*, particularly in pathogen recognition, where it activates the biosynthetic accumulation of NHP (N-hydroxy-pipecolic acid) at sites of infection. NHP is transported to distal tissues, triggering SAR, often accompanied by growth suppression, which ultimately leads to premature senescence (Bauer et al., 2021). In the present study, heterologous overexpression of *SiUGT26* in Arabidopsis was associated with delayed leaf senescence, as supported by rosette phenotype, Fv/Fm, and the reduced expression of *AtSAG12, AtSAG13, and AtSAG113.* In addition, the *SiUGT26*-GFP signal was observed in both the nucleus and cytoplasm. Because nuclear marker co-localization was not included in this study, the precise subcellular distribution pattern of SiUGT26 remains to be further confirmed. Research has demonstrated that WRKY proteins, a significant group of transcription factors, are key components of the dark-induced senescence transcriptome (Rivero et al., 2007). Transcriptomic studies further confirm the critical roles of WRKY transcription factors in regulating leaf senescence. These factors interact through activation, inhibition, or protein complex formation, thereby modulating downstream gene regulation (Majid and Abbas, 2019). In the present study, heterologous expression of SiUGT26 in Arabidopsis was associated with larger rosettes, higher Fv/Fm values, and reduced expression of selected senescence-associated marker genes. These measurements provide preliminary senescence-related evidence, but the representative rosette photograph alone does not demonstrate delayed senescence. The SiUGT26–GFP signal was observed in both the nucleus and cytoplasm; because a nuclear-marker co-localization assay was not performed, the precise subcellular distribution remains to be confirmed. Co-expression analysis further identified SiWRKY71 as a candidate associated with SiUGT26 expression. The observed correlation supports hypothesis generation but is not sufficient to demonstrate direct transcriptional regulation; promoter-binding, transcriptional-activation, and genetic analyses will be required to test this relationship.

Leaf size is an important component of plant architecture and light interception. In Arabidopsis, altered expression of NATsUGT73C6 and its associated UGT73C6 locus has been linked to rosette-size phenotypes (Meena et al., 2023). The enlarged rosettes observed in the SiUGT26-overexpressing lines therefore warrant separate consideration as a growth-related phenotype and should not be interpreted as stand-alone evidence of delayed senescence. The current functional evidence for SiUGT26 is limited to heterologous overexpression in Arabidopsis, rosette phenotype, Fv/Fm, and expression of selected senescence-associated marker genes. Additional physiological measurements, including chlorophyll content, ion leakage, ROS accumulation, and a defined senescence time-course analysis, would provide a more complete evaluation of the senescence process. Genetic validation in foxtail millet will also be required to establish the native function and enzymatic role of SiUGT26.

## Conclusions

This study systematically identified and characterized 173 UGT genes in foxtail millet. Phylogenetic classification, chromosomal distribution, duplication analysis, conserved motif and gene-structure analysis, promoter cis-element prediction, and expression profiling provide a genomic resource for future studies of the SiUGT family. Analysis of a published dark-induced leaf senescence transcriptome dataset together with regional RT-qPCR profiling identified SiUGT26 as a senescence-responsive candidate gene. Heterologous overexpression of SiUGT26 in Arabidopsis was associated with increased rosette size, higher Fv/Fm values, and reduced expression of selected senescence-associated marker genes, providing preliminary evidence of an association with senescence-related phenotypes. Co-expression analysis further identified SiWRKY71 as a candidate factor associated with SiUGT26 expression, although direct transcriptional regulation remains to be experimentally validated. Overall, this study provides a basis for future functional studies of UGT-associated leaf senescence responses in foxtail millet.

## Data Availability

The datasets presented in this study can be found in online repositories. The names of the repository/repositories and accession number(s) can be found in the article/**Supplementary Material**.
